# Postbiotics Unveiled: The Next Emerging Trend in Functional Foods

**DOI:** 10.1002/fsn3.72485

**Published:** 2026-10-14

**Authors:** Zoha Wahid, Abdul Momin Rizwan Ahmad, Farheen Zulfiqar, Syed Hassan Bin Usman Shah, Nilab Mohammadi

**Affiliations:** ^1^ Department of Human Nutrition and Dietetics NUST School of Health Sciences, National University of Sciences & Technology (NUST) Islamabad Pakistan; ^2^ Department of Food Science and Human Nutrition University of Veterinary and Animal Sciences Lahore Pakistan; ^3^ Department of Public Health PRIME Consulting Islamabad Pakistan; ^4^ Department of Curative Medicine Ghalib University Herat Afghanistan

**Keywords:** antimicrobial properties, bioactive compounds, functional foods, immunomodulatory properties, postbiotics, short‐chain fatty acids

## Abstract

The global population is expected to reach 9.7 billion by 2050, with demand for food increasing by up to 56%. Postbiotics are a safe and reliable substitute for probiotics, which are described as preparations of non‐viable bacteria and/or their metabolic byproducts. Data for this narrative review was collected using Goggle scholar and PubMed from 2006 to 2026. Major bioactive compounds such as short‐chain fatty acids, peptides, polysaccharides, vitamins, and cell fragments contribute to immunomodulatory, antioxidant, metabolic, and gut‐brain axis effects. Preclinical and clinical research shows that they can improve insulin sensitivity, control cytokine signaling, strengthen epithelial barrier integrity, and protect against obesity, type 2 diabetes mellitus, and depression. Postbiotics have also been found to improve mineral absorption and folate synthesis, and possess anti‐sarcopenic actions. Several commercial products are available because of their health benefits. Thus, postbiotics are a revolutionary development in microbiome‐based treatments; however, to completely understand their therapeutic potential, it is necessary to standardize, achieve mechanistic clarity, and ensure regulatory alignment.

## Graphical abstract

The graphical abstract shows different postbiotic production techniques, such as sonication, fermentation, heat inactivation, high‐pressure processing, dehydration, pH modification, and omic technologies. Additionally, it highlights the emerging health benefits and applications of postbiotics, including immune modulation, gut–brain axis regulation, metabolic health, antioxidant effects, anemia prevention, mineral balance, mental health, muscle strength, and various commercial producted produced using postbiotics.

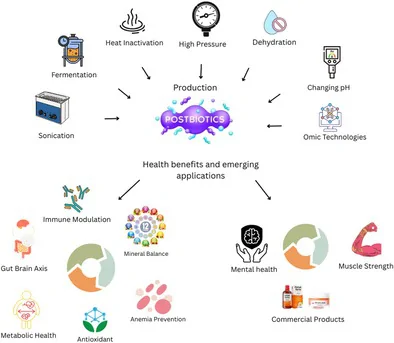

## 1 Introduction

The global population is estimated to rise to 9.7 billion people by 2050, resulting in a 21% increase in food demand from 35% to 56%. As a result, the probability of hunger has increased by 17% due to climate change (Khursheed et al. 2022). Traditional foods and herbal extracts have long been considered important elements of holistic care for overall health and well‐being. Delivered as functional foods or dietary supplements, nutraceuticals offer additional benefits beyond basic nutritional components. Numerous bioactive derivatives, including probiotics, postbiotics, fatty acids, amino acids, phytochemicals, and antioxidants, are present in them. They are all very beneficial to health (AlAli et al. 2021). According to market intelligence reports, the world's postbiotics market is expected to surpass 2 billion by 2030, showing increased consumer choice for safe and stable microbiome‐supporting products (Mafe et al. 2026).

The term postbiotics originated from the Greek language, “post,” meaning after, and “bios,” meaning life. Postbiotics are substances obtained from well‐defined, nonliving (inanimate, dead, or inactivated) microorganisms, whose preparation involves a delineated technological process that produces byproducts and inactivated live bacteria; they can also be reproduced. It also has substances produced by microbes, including metabolites, proteins, or peptides, that have a major role in the health benefits produced by a postbiotic; their metabolites are not crucial to a postbiotic (Vinderola, Sanders, and Salminen 2022).

Postbiotics are functional fermentation compounds that can be grouped into two commonly mentioned types: paraprobiotics and fermented infant formulas (FIFs). Paraprobiotics, also called ghost probiotics, are defined as non‐viable or deactivated microbial cells, which provide health benefits to the host when consumed in appropriate amounts. FIFs, in most cases, don't contain live bacteria and are present in infant or follow‐on formulas that undergo fermentation with lactic acid‐producing or some other bacteria (Wegh et al. 2019). Probiotics include living beneficial microorganisms either found in the gut or used in food products due to their positive health benefits. Safe and equally effective health benefits are provided by the bacterial products and probiotic components termed as “postbiotics” (Scarpellini et al. 2021). Postbiotics are most commonly produced from Lactic Acid Bacteria (LAB); their production involves bioactive, soluble metabolic byproducts that are obtained from food‐grade microorganisms during their growth process and fermentation in complex microbiological cultures, food, or the gut. These compounds are produced by the action of microorganisms on food ingredients (Moradi et al. 2020). Modulation of complex microbial environments and interaction with immune cells inhabiting surfaces confer targets for mitigating health. Food fermentation, commonly by LAB, produces several byproducts, including short‐chain fatty acids (SCFAs), which exert a beneficial impact on health (Salminen et al. 2021).

Postbiotics are also called biogenics, metabiotics, or simply metabolites/CFS (cell‐free supernatants). They are soluble compounds that are either generated by living bacteria or released resulting from bacterial lysis. These include enzymes, peptides, teichoic acids, peptidoglycan‐derived muropeptides, endo‐ and exo‐polysaccharides, cell surface proteins, vitamins, plasmalogens, organic acids, and short‐chain fatty acids (SCFAs) (Aguilar‐Toalá et al. 2018). Since postbiotics are more stable and do not have to be handled, preserved, or transported under tight conditions like a cold chain, they can be a great substitute to be used in commercial products. Although there are not many studies on postbiotics in food, inactivated LAB bacteria can be found in foods like yoghurt and sports drinks made with whey. *L. bulgaricus, Streptococcus thermophilus, Lactobacillus acidophilus, Lactobacillus gasseri* CP2305, and *Lactobacillus casei* 01 are a few examples that have already been studied (Vera‐Santander et al. 2023). Numerous fermented foods, including yoghurt, sauerkraut, pickled vegetables, and kombucha, naturally contain postbiotics. These are made by cell disruption techniques like heating, high pressure, enzymatic treatment, co‐culturing, solvent extraction, chemical treatment (like formalin), and sonication of different bacterial and fungal species (Thorakkattu et al. 2022).

Postbiotics are considered an effective tool in combating human diseases, according to growing evidence. Numerous clinical and animal studies have shown that postbiotics can improve dental health, protect against osteoporosis, boost immunity, and combat allergies and colorectal cancer (CRC) (Dong et al. 2024). Postbiotics are essential for immune system regulation, strengthening the epithelial barrier, and regulating the protective function against infections. They are thought to enhance vaccine effectiveness and are utilized extensively in food, pharmaceutical, and biomedical industries (Ma et al. 2023). Postbiotics are an alternative to traditional antibiotics and food preservatives because they are nonviable and can successfully stop the growth of foodborne infections. Because of their antioxidative qualities, they are essential in preventing long‐term conditions like diabetes, cancer, and heart disease (Isaac‐Bamgboye et al. 2024).

The aim of this review is to clarify the terminology and trace how the concept of a postbiotic evolved from a by‐product of fermentation into a defined class of bioactive preparation; second, to map production and characterization methods against the bioactive compounds they yield, so that manufacturing choices can be linked to functional outcomes; and third, to critically appraise the evidence for health effects across immune, gut–brain, metabolic, antioxidant, hematological, and mineral‐related endpoints, distinguishing throughout between in vitro, animal, and human findings. The novelty of the review lies less in cataloguing individual studies than in connecting the production pipeline to physiological effect, and in being explicit about where the evidence is strong, where it rests largely on animal or cell models, and where claims have outrun the data.

## 2 Methodology

### 2.1 Data Sources and Search Strategy

This narrative review presents literature on postbiotics, their production, and their functional and health‐related applications. Two electronic databases, PubMed and Google Scholar, were searched from inception to 2006, and the search was last refreshed in 2026. PubMed provided peer‐reviewed biomedical indexing, while Google Scholar was used to capture a wider and more interdisciplinary body of work, including food‐technology and animal‐nutrition literature that is not always indexed in PubMed. The database searches were supplemented by hand‐searching the reference lists of key reviews and consensus statements, which brought in several relevant studies that the keyword searches had missed.

Because the vocabulary of this field is inconsistent, the search deliberately combined a broad set of synonyms for the intervention with terms for its sources and effects, linked by the Boolean operators AND and OR and using truncation to catch spelling and word‐ending variants. The full search string and the filters applied are set out in Table S1. In brief, intervention terms such as “postbiotic,” “paraprobiotic,” “biogenics,” “metabiotic,” “microbiota metabolites,” “cell‐free supernatant,” and “heat‐killed/inactivated bacteria” were combined with outcome and context terms including “functional food,” “health benefit,” “immunomodulation,” “short‐chain fatty acid,” “gut–brain axis,” “metabolic disorder,” “production,” and “standardization,” with truncation applied to each root term. For the production dataset compiled in Table S2, a further filter restricted results to original research (experimental) articles, so that production media, processing steps, and reported bioactive outcomes were drawn from primary data rather than from secondary summaries. Health effects of postbiotics by system: proposed mechanisms, level of evidence, and representative studies are given in Table S3.

### 2.2 Eligibility Criteria

Articles were eligible if they were written in English, published in peer‐reviewed journals, and reported on the definition, production, characterization, mechanisms, or health effects of postbiotics or closely related preparations (paraprobiotics, inactivated microbial cells, and cell‐free metabolic fractions). In vitro, animal, and human studies were all admitted, and both human and animal nutrition contexts were retained so that the review would reflect the true breadth of the current evidence. Preference was given to work published from 2018 onward, the period over which the modern definition took shape, but earlier foundational and mechanistic papers were kept where they remained scientifically relevant.

Records were excluded if they were not in English; if they were available only as conference abstracts, preprints, theses, editorials, or opinion pieces lacking primary data; if they dealt exclusively with live probiotics and contained no postbiotic, paraprobiotic, or inactivated component; or if they duplicated a study already included. For the production entries in Table S2 specifically, review articles were not accepted as primary sources, since only original experimental studies could provide verifiable production and characterization parameters. This last criterion directly addresses the earlier reliance on review articles for production data and explains the replacements made in that table.

### 2.3 Study Selection, Quality Appraisal, and Synthesis

Titles and abstracts of the retrieved records were screened for relevance, and the full texts of the remaining articles were then assessed against the criteria above. Because the review is descriptive and integrative rather than quantitative, the evidence was synthesized narratively and organized thematically by application area: immune modulation, the gut–brain axis, metabolic disorders, antioxidant activity, anemia, mineral modulation, and emerging uses rather than pooled in a meta‐analysis.

As is appropriate for a narrative review, a formal risk‐of‐bias or GRADE‐style quality assessment was not undertaken. Instead, the strength of each body of evidence was weighed qualitatively: controlled human trials and reproducible animal studies were given more interpretive weight than isolated in vitro observations, and single‐strain, single‐study claims are identified as such in the text. This approach enabled in assessing the reliability of findings without implying a level of methodological uniformity that the underlying studies do not share.

## 3 Evolution of Postbiotics: From Byproducts to Bioactive Powerhouses

Our gut microbiome basically produces postbiotics as byproducts. Postbiotics are also produced following the digestion of probiotic and prebiotic meals (Bhuyan et al. 2023). In 2021, a panel of experts established by the International Scientific Association of Probiotics and Prebiotics published a definition and scope for the term postbiotic: “Preparation of non‐living microorganisms and/or their constituents that impart health advantages to the host” (Vinderola, Sanders, Salminen, and Szajewska 2022). The terms “postbiotics” and “paraprobiotics” were frequently used interchangeably in research before the international scientific association for probiotics and prebiotics (ISAPP) definition of postbiotics. In the context of probiotic metabolism, metabiotics are defined as purified bioactive substances with established molecular mechanisms, while paraprobiotics are inactivated or killed microbial cells (Nataraj et al. 2020). The phrase “postbiotics” originated in the field of probiotics research. They are conventionally regarded as metabolic byproducts of probiotic activity, along with bioactive substances that have distinct health benefits, such as enzymes, peptides, teichoic acids, polysaccharides, cell wall fragments, and organic acids generated during microbial fermentations (Amiri et al. 2021). Postbiotic preparation of yeast culture produced via biological fermentation technology is found to be high in organic acids, nucleotides, β‐glucans and other functional components (Chalotra et al. 2026). Figure 1 shows various production techniques involved in postbiotic manufacturing. In order to separate paraprobiotics and postbiotics from various probiotic microbes, cell disruption procedures such as sonication, high pressure, ionizing and UV radiation, solvent extraction, heat treatment, and enzymatic treatments are used.

**FIGURE 1:**
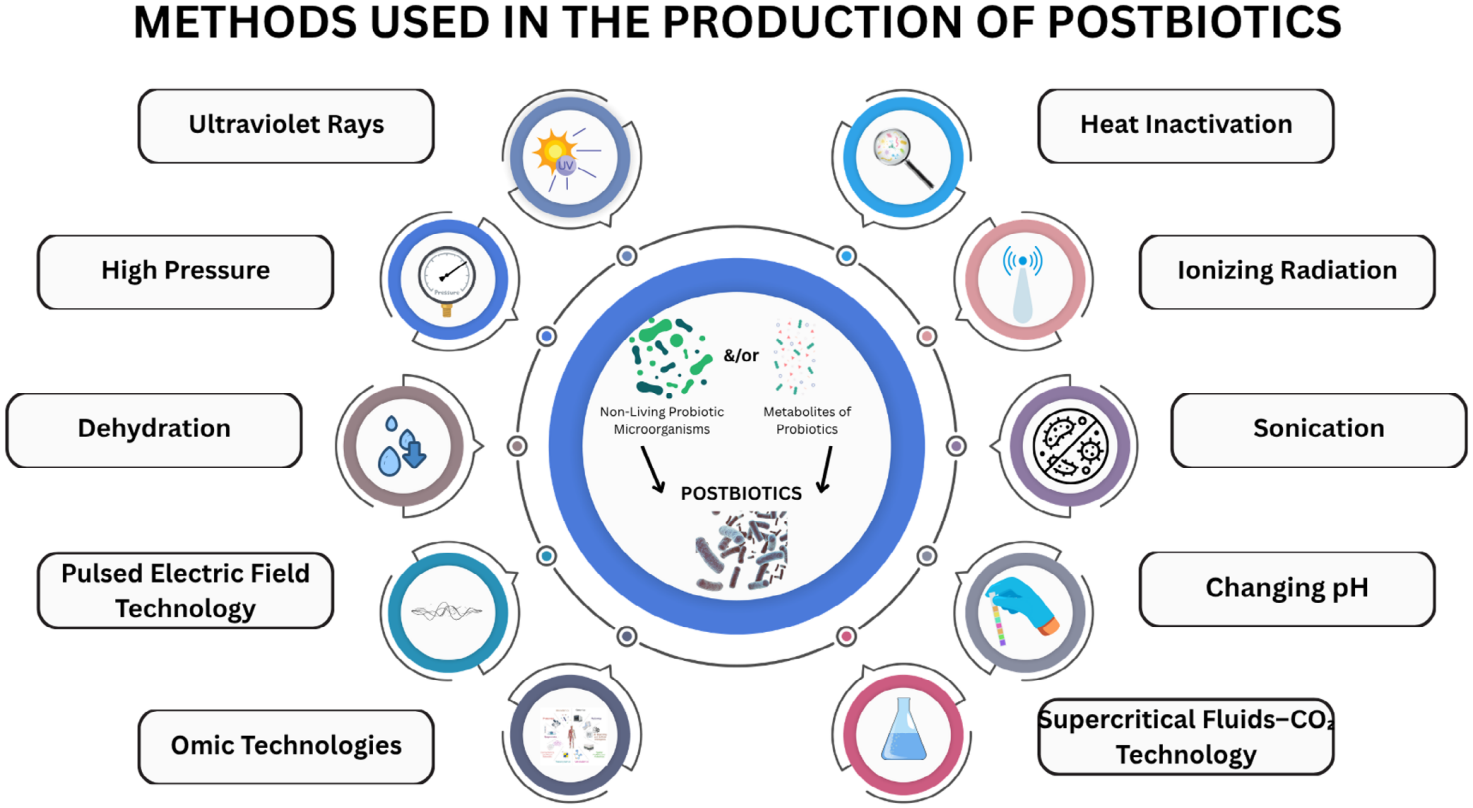
Production techniques involved in postbiotic manufacturing, such as microbial fermentation followed by cell disruption methods such as sonication, high‐pressure processing, ionizing and ultraviolet (UV) radiation, solvent extraction, heat treatment, and enzymatic treatments for the generation of paraprobiotics and postbiotics.

Many additional techniques, including ohmic heating, supercritical CO_2_, drying, pulsed electric field (PEF), and pH adjustments, may also be employed for the manufacture (Teame et al. 2020). High‐performance liquid chromatography (HPLC), high‐field proton nuclear magnetic resonance spectroscopy (1H‐NMR), matrix‐assisted laser desorption/ionization time‐of‐flight (MALDI‐TOF) mass spectrometry, or liquid chromatography mass spectrometry (LC/MS and LC/MS/MS) for both qualitative and quantitative evaluation are methods used to further identify postbiotic components (Mayorgas et al. 2021).

The types of postbiotics produced depend on the manufacturing process and microbial inactivation; quantification methods applied should be based on the application, such as for research, manufacturing process control, regulatory compliance, or consumer labeling. For measurement of intact, inanimate cells, the major method used is flow cytometry; it is cheap and requires less instrumentation. Quantitative PCR and digital PCR are also employed. For analysis of cell fragments and metabolites, a list of chemical, biochemical, microbiological, and immunological assays are carried out (Vinderola et al. 2025). Varied composition and product‐specific production requirements pose challenges in postbiotic development. Tailored processing is required for the production of different classes of postbiotic metabolites, and in turn, their bioactivity is dependent on manufacturing conditions. Large‐scale commercialization is affected by limited analytical standardization, a poor regulatory framework, and economic constraints (Balakrishna et al. 2026). Detailed production media, processes, and their final products obtained from various studies are shown in Table 1.

**TABLE 1:** Microbial species, production methods, stabilization techniques, and associated bioactive outcomes in postbiotic development.

Microbial species	Production medium	Production process	Stabilization technique/analytical techniques	Quality of end product	References
*Lactiplantibacillus plantarum* PTCC1745	MRS broth, cheese whey, and low‐fat UHT milk (1.5% fat)	Fermentation at 37°C for 48 h; cultures divided into heat‐treated (95°C, 5 min), sonicated (5 min), and intact groups; centrifugation (4200×*g*, 10 min, 4°C); filtration through 0.45 μm filter	Stabilization: Heat treatment, sonication, centrifugation, filtration, storage at 4°C. Analytical techniques: MIC/MBC microdilution assay, agar well diffusion antibacterial and antifungal assays, dynamic light scattering (DLS) for particle size, ANOVA (GraphPad Prism)	Cell‐free postbiotic supernatant (nanoparticle‐sized postbiotic preparation)	Khakpour et al. (2024)
*Lactobacillus fermentum BGHV110*	MRS broth (37°C, anaerobic conditions using Anaerocult A)	Human isolate identified by 16S rDNA sequencing. Overnight culture was pelleted (5000 rpm, 10 min), washed twice with PBS, concentrated 10‐fold in PBS, disrupted by French press (3 passages) to obtain a bioactive lysate (postbiotic)	**Stabilization:** Lyophilization (freeze‐drying) and storage at +4°C. **Analytical techniques:** 16S rDNA sequencing, PCR, MTT assay, LDH cytotoxicity assay, Western blotting (LC3, p62), Bradford protein assay, qRT‐PCR (BECN1, ATG5, PINK1, p62/SQSTM1), ImageJ densitometry, SPSS statistical analysis	Lyophilized postbiotic (HV110) retained biological activity. 3 mg/mL was identified as the optimal non‐cytotoxic dose. HV110 significantly protected HepG2 cells from acetaminophen‐induced hepatotoxicity by improving cell viability, reducing cytotoxicity, inducing autophagy (increased LC3‐II/LC3‐I ratio and BECN1 expression), activating the PINK1 pathway, and enhancing cytoprotective effects without causing toxicity at the selected dose	Dinić et al. (2017)
*Lactobacillus amylovorus* CP1563	MRS broth (37°C); passaged three times in food‐grade medium before fragmentation	Cells harvested by centrifugation, washed with Milli‐Q water, heat‐killed (90°C), mechanically disrupted using DYNO‐MILL (also compared with jet mills and planetary mills) to produce fragmented bacterial cells (paraprobiotic). Organic extracts prepared by ethanolic KOH saponification followed by diethyl ether extraction for PPAR ligand assays	**Stabilization:** Lyophilization of fragmented bacterial cells. **Analytical techniques:** Luciferase reporter assays for **PPARα/PPARγ**, scanning electron microscopy (SEM), WinROOF image analysis, animal feeding studies (high‐fat diet mice), plasma lipid analysis (HDL, LDL, TG), ELISA (adiponectin), CT scanning for visceral fat, statistical analysis (two‐way ANOVA, Dunnett's test)	Fragmented paraprobiotic demonstrated strong PPARα/PPARγ agonist activity, increased HDL cholesterol, decreased LDL cholesterol, triglycerides and atherosclerosis index in high‐fat diet‐fed mice. Greater fragmentation enhanced biological activity, indicating improved release of bioactive lipid ligands	Nakamura et al. (2016)
*Lactiplantibacillus plantarum* 299v; *Lacticaseibacillus casei* Shirota; *Bifidobacterium animalis* subsp. *lactis* BPL1	MRS broth; Control whey (CW); Whey +1% inulin (WIn1%); Whey +2% inulin (WIn2%); Whey +1% chia mucilage (WMc1%); Whey +2% chia mucilage (WMc2%)	Fermentation at 35°C for 30 h → Centrifugation (7000×*g*, 15 min, 5°C) → 0.45 μm membrane filtration → Cell‐free supernatant (postbiotic) collected and stored at −18°C	HPLC (lactic acid & SCFAs); Folin–Ciocalteu assay (total phenolics); DPPH radical scavenging assay; KMnO_4_ agar antioxidant assay; pH; Titratable acidity; Viable cell count/growth kinetics (Modified Gompertz model)	Cell‐free postbiotic (CFS); Lactic acid; Acetic acid; Propionic acid; Butyric acid (SCFAs); Total phenolic compounds; Antioxidant activity	Vera‐Santander et al. (2024)
*Bacillus amyloliquefaciens* J + *Lactiplantibacillus plantarum* SN4	Corn flour, soybean meal, peptide powder, wheat bran supplemented with glucose, molasses, growth factor, CaCO_3_, yeast extract and KH_2_PO_4_ (optimized formulation)	Solid‐state fermentation (SSF); mixed culture (1:1); optimized conditions: 37°C, 8 days, 8% inoculum, 50% moisture; optimized medium containing 8.82% glucose, 30.02% corn flour and 28.57% soybean meal	**Stabilization:** Water extraction, centrifugation, 0.22 μm membrane filtration, freeze‐drying (lyophilization), storage at −20°C. **Analytical techniques:** HPLC (lactic acid, phenyllactic acid, ferulic acid), GC (SCFAs), BCA protein assay, Folin–Ciocalteu assay, phenol–sulfuric acid assay, MIC/MBC determination, DPPH, ABTS and hydroxyl radical scavenging assays, reducing power assay, CCK‐8 cell viability assay, Griess NO assay, wound‐healing assay, Design‐Expert and SPSS statistical analysis	Freeze‐dried postbiotic powder rich in lactic acid, 3‐phenyllactic acid, ferulic acid, short‐chain fatty acids (acetic, butyric, propionic and pentanoic acids), phenolic compounds, soluble sugars and proteins, with antimicrobial, antioxidant, anti‐inflammatory and wound‐healing properties	Tong et al. (2023)
*Lactobacillus plantarum* AC131	Modified MRS (mMRS) broth supplemented with DDGS hydrolysates (reducing sugars) and/or 10% rose wastewater (Rosa alba/Rosa damascena); control MRS broth	Fermentation in Erlenmeyer flasks and Bioflo bioreactor (37°C; with and without pH control) using acid‐ and enzyme‐hydrolyzed DDGS as carbon source	HPLC (lactic acid & reducing sugars), spectrophotometric biomass measurement (OD620), agar well diffusion assay (antimicrobial activity), Crystal Violet biofilm assay, Bertrand's method (reducing sugars)	Lactic acid; biomass; antimicrobial postbiotics (cell‐free supernatant); bioactive metabolites; parabiotics (heat‐killed cells)	Danova et al. (2023)
*Lactiplantibacillus plantarum* F2, *Pediococcus pentosaceus* 50, *Leuconostoc mesenteroides* AF1	MRS broth	Centrifugation (4000×*g*, 10 min, 4°C) and filtered using 0.45 μm membrane filer; complete heat treatment at 80°C for 30 min; viability confirmed by culture on MRS agar	Refrigerated (4°C) or −20°C storage; antioxidant activity (DPPH assay); total phenolic content (Folin–Ciocalteu assay); LC–MS/MS for phenolic compounds; HPLC for organic acid profiling	Organic acids (lactic acid, acetic acid, citric acid, succinic acid, tartaric acid), phenolic compounds (chlorogenic acid, caffeic acid, vanillic acid, luteolin), antimicrobial metabolites	Almahbashi and Gunes Altuntas (2025)
*Bifidobacterium animalis subsp. lactis* HN019	Optimized semi‐defined medium (Medium 4) containing glucose (30 g/L), yeast extract (20 g/L), soy peptone (15 g/L), phosphate salts, ammonium citrate, Tween 80, L‐cysteine and trace minerals	Batch, fed‐batch and in situ product removal (ISPR) fermentation in 3–22 L bioreactors at 37°C, pH 6.2, 150 rpm. Biomass concentrated by microfiltration; EPS recovered from fermentation supernatant	Stabilization: Microfiltration, diafiltration, spray drying with trehalose: sucrose (1:1) for probiotic biomass; freeze‐drying after heat inactivation for postbiotic broth. Analytical techniques: HPLC, SEC‐TDA, GC–MS, ^1^H‐NMR, Phenol–Sulfuric Acid assay, Western blot, Immunofluorescence (ZO‐1), ELISA (IL‐6), viability assay (CFU), dry cell weight measurement	ISPR achieved 2.9 × 10^10^ CFU/mL (highest biomass yield). Spray‐dried biomass retained 8.7 × 10^10^ CFU/g and remained stable for 12 months at 4°C. Purified EPS showed ~87%–88% purity, molecular weight 32.3 kDa, predominantly glucose (94.5%), reduced TLR‐4/NF‐κB activation, restored ZO‐1 expression and decreased IL‐6 secretion in LPS‐treated Caco‐2 cells	D'ambrosio et al. (2024)
*Bifidobacterium animalis* subsp. *lactis* BB12	Food‐grade cultivation medium based on cheese whey or milk permeate supplemented with yeast extract and linoleic acid—optimized conditions: pH 5, 2.5% yeast extract, 38°C	Fermentation in 200 mL Erlenmeyer flasks containing 50 mL medium inoculated with 1 × 10^9^ CFU/mL. Optimization performed using factorial design and Box–Behnken response surface methodology	Viable cell count (RCM agar), UV–Vis spectrophotometry (CLA), Phenol–sulfuric acid assay (EPS), Agar well diffusion assay (BACs), FTIR, GC‐FID, TLC, SDS‐PAGE	Conjugated linoleic acid (CLA), exopolysaccharides (EPS), bioactive compounds (BACs) with antimicrobial and antioxidantproperties	Amiri et al. (2021)

*Note:* This table shows different microbial species used in postbiotic production with varied fermentation conditions, processing techniques, and stabilization approaches and their subsequent effect on the bioactive profile of the final product.

## 4 Nutritional Profile of Postbiotics

A diverse group of bioactive compounds is released during fermentation or microbial inactivation, such as cell‐free supernatants, exopolysaccharides, enzymes, cell wall fragments, short‐chain fatty acids, bacterial lysates, and gut microbiota‐derived metabolites; each component possesses a distinct therapeutic role and biological activities. Several advantages are attributed to postbiotics over probiotics, such as greater production, long shelf life, and reduced storage requirement; as well as from a safety perspective, it reduces the risk linked with live microbe administration, which can in turn lead to antibiotic resistance (Żółkiewicz et al. 2020). Products that have significant nutritional and biological value can be consumed for better population nutrition. Around 95%–97% of postbiotics remain stable and reach unchanged. Postbiotics have a positive impact on the immune system, anticancer, and cholesterol‐lowering effects in addition to optimizing the gastrointestinal tract's function (Rozhkova et al. 2023). Short‐chain fatty acids (SCFAs), including acetic, propionic, and butyric acids, and vital minerals like vitamin K, B vitamins, and aromatic amino acids, are a few of the most well‐known postbiotic metabolites derived from probiotics. Glutathione: obtained by *Lactobacillus fermentum* ME3, antimicrobial peptides (AMPs) that fall within the defensin and bacteriocin categories, phytoestrogens: equol, enterolactone, enterodiol, urolithin A and urolithin B, hydrogen peroxide, phenolactic acid, D‐amino acids, and fulvic acid (Pelton 2020).

Certain postbiotics, including lignans (enterodiol and enterolactone), ellagitannins (primarily Uro‐A), isoflavones (equol and to a lesser extent, O‐desmethylangolensin, ODMA), and hop prenylflavonoids (8‐prenylnaringenin, 8PN), are derived from polyphenols and have numerous medicinal properties (Cortés‐Martín et al. 2020). The *Lactobacillus* species, *Paenibacillus polymyxa* , and lactic acid *bacilli* produce a variety of homo‐ and hetero‐polysaccharides, including uronic acid, β‐glucans, and kefricin (Aggarwal et al. 2022).

Lantigen B, the first bacterial lysate to be prepared by alkaline extraction, has in vivo effects through a variety of mechanisms, including mechanical lysate generation using the most widely sold representative brand of mechanical lysate preparations. Polyvalent mechanical bacterial lysate (PMBL) is found in Ismigen, while ribosomal‐rich lysates are another kind of mechanical lysate (Suárez et al. 2020). During the late log or start of the stationary phase of the growth cycle, a variety of polymeric molecules known as biosurfactants (BS) are created. A few examples of these include glycolipids, lipopeptides, phospholipids, neutral lipids, polysaccharide‐protein complexes, and free fatty acids that are extracted using a solvent extraction principle (Nataraj et al. 2020).

Postbiotics made from probiotic bacterial strains belonging to the *Lactobacillaceae* and *Bifidobacterium* families contain live microbial cells as well as a mixture of metabolites called cell‐free culture supernatants (CFS), which are released into the culture medium along with cell structural components when cells are lysed or inactivated. These substances also have several health advantages (Rocchetti et al. 2024). Teichoic acids, different peptidoglycan fragments generated through the activity of endogenous peptidoglycan hydrolases, and bacterial pili are additional parts of bacterial cell walls that have been identified to play an immunological role (Aggarwal et al. 2022).

The postbiotics produced by *L. casei* CRL 431 and *Bacillus coagulans* GBI‐30 were separated into intracellular and cell wall fractions, and both were rich in fatty acids, amino acids, and other metabolites. It was also discovered that the quality of the postbiotics produced is greatly influenced by the processing method. Increased biological activity and bioavailability of postbiotics were demonstrated by Raman spectroscopy and in silico analysis (Aguilar‐Toalá et al. 2020).

Lactic, acetic, succinic, and other essential and non‐essential organic acids were found to be present in postbiotic compounds derived from *Lacticaseibacillus paracasei* ABK and *Lactobacillus helveticus* H9. Free amino acids and organic acids, including lactic, acetic, and succinic acids, significantly increased as a result of the fermentation of a dairy‐based source (reconstituted skim milk). While MRS broth only significantly increased methionine in *L. paracasei* ABK, it showed less change overall (Rozhkova et al. 2023). According to research, postbiotics generated from LAB in plant‐based sourdough fermentation provide a naturally occurring bioactive material that is highly stable during processing. By decreasing phytic acid, improving the bioavailability of minerals (such as calcium, iron, and zinc), and enriching bakery goods with SCFAs, B‐vitamins, and phenolic compounds, sourdough fermentation further enhanced bakery products' nutritional profile (Asqardokht‐Aliabadi et al. 2025).

Postbiotics derived from honey bee pollen from *Weissella confusa* and *W. cibaria* are abundant in fatty acids (stearic and caprylic acids), organic acids (lactic and formic acids), water‐ and fat‐soluble vitamins (B‐complex and E), and simple carbohydrates (glucose, fructose) (Kiran et al. 2023). Increased levels of B vitamins, lysine, protein fractions, sugar, and soluble dietary fiber are produced when uncooked or baked cereals undergo fermentation. It has a malty, mildly sweet, and sour taste that boosts mineral absorption. Additionally, it has been shown to have the capacity to decrease toxic gliadin peptides in gluten and produce advantageous substances like GABA and ACE‐inhibitory peptides. Because of their ability to inhibit phytate and mold, they have also been used to ferment grains (Wegh et al. 2019). Figure 2 shows detailed classification and interactions of biotics: prebiotics, probiotics, postbiotics, and symbiotic.

**FIGURE 2:**
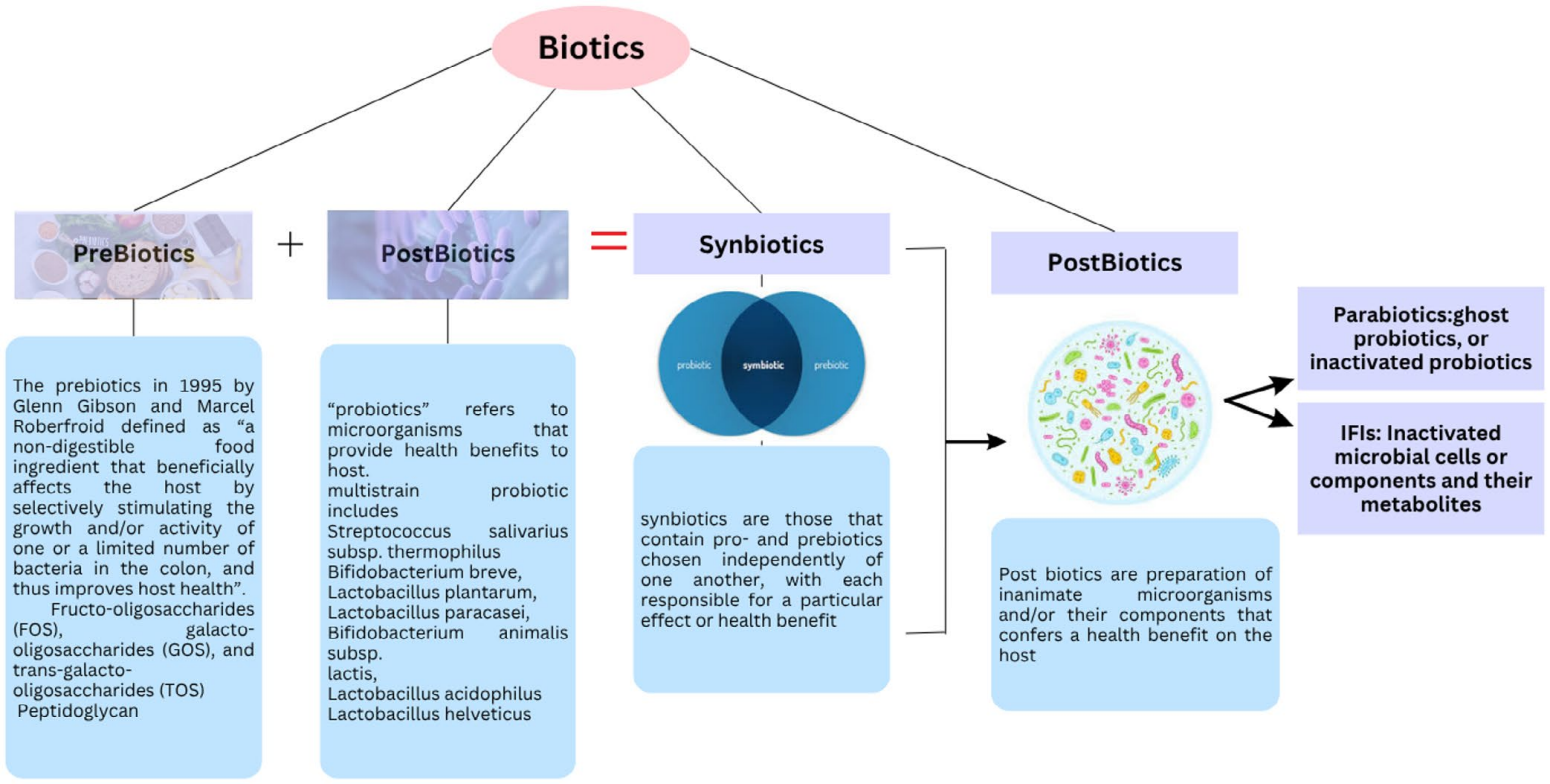
Classification and interactions of biotics, including prebiotics, probiotics, postbiotics, and synbiotics, and their functional roles and synergistic relationships in gut microbiota modulation and host health.

## 5 Regulatory Perspective on Postbiotics

ISAPP panel proposed the term postbiotics, constituting a prefix “post” meaning “After” and “biotics” meaning “relating or resulting from life”; as a whole, it is defined as “preparation of inanimate microorganisms and/or their components that confer a health benefit on the host.” The words chosen fit well in elaborating the core concept, such as “inanimate” meaning lifeless, rather than inactive; “preparations” reflects the likelihood that specific microbial biomass formulations, matrices, and/or inactivation is used (Salminen et al. 2021). Studies show that regulations for the postbiotic production and use as dietary supplementation are not present due to their complexities. FDA in Thailand authorized the use of heat‐killed bacteria as a dietary supplement, whereas no official regulations have been presented by the Chinese government. NDIs have been submitted by the US for postbiotics; no objection has been raised by Health Canada on the term postbiotics. However, in Europe, postbiotic ingredients need to follow the regulatory claims so that they can be used in foods and supplements, as the postbiotic term is not regulated (Guglielmetti et al. 2025). Several non‐viable microbial products have been developed and used in several European countries between the 1980s and today that fit completely on ISAPP's definition of postbiotics, such as for the management of diarrhea in children, heat‐inactivated *Limosilactobacillus fermentum* CNCM MA65/4E‐1b plus *Lactobacillus delbrueckii* subsp. *delbrueckii* CNCM MA65/4E‐2z are produced along with their fermentation metabolites (Malagón‐Rojas et al. 2020). According to the ISAPP definition, postbiotics can be produced directly from non‐viable microorganisms. Developers rely on existing regulations for food and dietary supplements, as there are no dedicated regulations for postbiotics. However, postbiotics may be considered as biological medicinal products under the European Union pharmaceutical framework (Directive 2001/83/EC and 2004/27/EC) (Vinderola et al. 2023).

EU novel food authorization 2021 for pasteurized *Akkermansia muciniphila* to be used as food for medical purposes indicated that the inanimate form of the microbe had beneficial properties in human clinical studies. It is used under EU Directive 2002/46/EC and Regulation (EU) No 609/2013 in food supplements and food for medicinal purposes (Cani et al. 2022; Vinderola et al. 2023). The production and market for pre‐, pro‐, and postbiotics is evolving in India; to ensure safety and efficacy, these products are regulated under the Food Safety and Standards Authority of India (FSSAI). Microbial safety and validity are crucial in probiotics, and postbiotics is essential throughout the product's shelf life (Bhattacharjee et al. 2025).

Definition and regulatory pathways for postbiotics are yet to be provided by agencies like the US FDA and EFSA. Literature shows that, unlike probiotics, postbiotics come under general categories like food additives, microbial metabolites, or novel food ingredients (Urban et al. 2024). EFSA categorizes postbiotics and novel foods that require detailed safety and efficacy data before approval (Precup et al. 2024). Moreover, certain postbiotic‐derived ingredients have been recognized by FSA's GRAS framework (Spacova et al. 2023). Recent studies show variation in regional trends; functional efficacy and compositional stability evidence are required by China NHFA, Food and Function claims in Japan are regulated as functional foods, and risk‐based assessment is applied by EU (Mafe et al. 2026).

Literature involving randomized controlled trials on 1740 infants showed that postbiotics prove to be beneficial for prevention and management of infectious diseases in children under 5 years. However, a few clinical studies constituting supplementation of heat‐inactivated *L. acidophilus* with micronutrients showed mild side effects such as abdominal distention, dehydration, and vomiting (Asif et al. 2023).

## 6 Role of Artificial Intelligence in the Discovery of Postbiotics

Transformation in postbiotic discovery is brought by artificial intelligence (AI), by integrating metabolomic, microbiomic, multiomic datasets, dietary patterns, and health status for identification and optimization of bioactive components. AI can assist in the development of targeted postbiotic products with improved health benefits by optimizing microbial strain selection, metabolite profile, and formulation design. AI can help in safe prediction of metabolic response and potential risk in preclinical safety evaluation trials through in silico toxicological screening and host microbiome interaction (Bhuiyan et al. 2026). Previously undiscovered bioactive compounds can be predicted using AI‐based platforms such as antiSMASH, PRISM4, and DeepBGC, which help in biosynthetic gene cluster detection responsible for secondary metabolite production. AI also helps in functional characterization of postbiotics; AlphaFold2 can provide molecular insights about protein function by accurately predicting the three‐dimensional amino acid structure (Behl and Sharma 2026).

Research shows that an AI‐driven approach can be used to understand the specific indole effects in indole postbiotic therapy. Integration of host data and microbiome can support personalized therapy; it may also be used to optimize formulation, predict pharmacokinetics, and identify bioactive compounds. This aims to translate the findings from experimental models to clinical products that can be used to provide specific health benefits (Matteo et al. 2026). Furthermore, real‐time monitoring of the fermentation process along with the prediction and control of microbial dynamics can be performed by integration of AI with biosensors and Internet of Things (IoT)‐based smart fermentation platforms. Classification of raw materials, fermentation process monitoring, and prediction of volatile metabolites through sourdough fermentation can be successfully carried out by integrating machine learning models with analytical techniques (Nguyen and Nguyen 2025). Research shows that AI‐driven platforms can be used to predict a specific postbiotic compound and the respective dose that can be beneficial for an individual based on gut ecology and clinical phenotypes. However, the use of AI for individualized recommendation can pose several issues such as data privacy, algorithmic transparency, and equitable access to decision‐support tools (Mafe et al. 2026).

## 7 Health Benefits

### 7.1 Immune Health

The derivatives of probiotic microorganisms, which are nonviable and called postbiotics, have attracted attention as promising immunomodulatory agents. In contrast to probiotics, which require viability to provide their functions, postbiotics are safer, more consistent, and stable especially in vulnerable populations like neonates and individuals with weak immune systems (Aguilar‐Toalá et al. 2018; Salminen et al. 2021). Teichoic acid is a polysaccharide derivative and is divided into two major types: lipoteichoic acid and wall teichoic acid. Studies show that teichoic acids from *L. rhamnosus* GG boost immunity and lower inflammation, and lipoteichoic acid from *Lactiplantibacillus plantarum* MTCC 5690, *L. fermentum* MTCC 5689, and *L. rhamnosus* GG also modulate the immune system (Amobonye et al. 2025).

They also act as immune‐modulators and do so via complicated interactions (with microbial‐associated molecular patterns (MAMPs), immune cell communication, cytokine regulation and epithelial fortification). The immunomodulation potential of postbiotics is triggered by their potential to interact with pattern recognition receptors (PRRs), that is, Toll‐like receptors (TLRs) and NOD‐like receptors (NLRs) via their MAMPs, for example, peptidoglycans, lipoteichoicacids (LTAs) and surface‐layer proteins (SLPs) (Behzadi et al. 2021). This pro‐inflammatory surge is usually temporary and is contrasted by a regulatory phase with release of anti‐inflammatory cytokines, for example, IL‐10 and TGF‐b which will assist in immune resolution and tolerance (Roberts et al. 2024).

In one example, SLP‐A of *L. acidophilus* is found to bind to C‐type lectin receptors of dendritic cells causing differentiation of regulatory (Treg) cells and leading to immune tolerance (Konstantinov et al. 2008). Moreover, studies demonstrate that postbiotic components can enhance antigen presentation and the expression of co‐stimulatory molecules on immune cells, thus supporting effective T‐cell responses (Nataraj et al. 2020).

Among important postbiotics roles is the capacity to reinforce the epithelial barrier. *Lactobacillus rhamnosus* GG secretes such proteins as p40, p75, and HM0539, which stimulate the EGFR signaling pathway and protect the tight junction protein without letting bacteria pass through (Bingöl et al. 2024). Heat‐inactivated *Bifidobacterium longum* CECT‐7347 also improved gut barrier integrity and the immune responses in vivo studies (Martorell et al. 2021).

Short‐chain fatty acids (SCFAs), butyrate, acetate, and propionate, belong to metabolites possessing postbiotic activity and are an essential part of immune regulation. The mechanism through which SCFAs regulate Treg cell differentiation and inhibit Th17‐driven inflammation is that SCFAs inhibit histone deacetylase (HDACs), thereby altering gene expression (Parada Venegas et al. 2019). However, butyrate specifically increases epithelial integrity in an IL‐10 receptor‐dependent process, which antagonizes inflammation in colitis models (Zheng et al. 2017). Other metabolites with antimicrobial effects, like bacteriocins and organic acids, also contribute to maintaining mucosal immunity by pore formation leading to cell lysis and by preventing cell wall synthesis. Bacteriocins produced by Bifidobacterium are found to inhibit antibiotic‐sensitive and resistant strains of *H. pylori* (Sarkar and Mandal 2016).

Clinical studies facilitate the immunotherapeutic prospect of postbiotics. Tyndallized *Lactobacillus rhamnosus* has proved effective in alleviating symptoms of atopic dermatitis, decreasing serum IgE, and controlling Th2 disease (Lee et al. 2016). Similarly, heat‐killed *Lactobacillus paracasei* was found to be effective against common cold in geriatric groups, which makes it a potential vaccine adjuvant (Akatsu 2021). These effects underscore postbiotics' utility in both allergic and aging‐associated immune dysfunction. Safety of postbiotics and technological strength represent one of their essential health benefits. They are not viable, which rules out the risks of microbial translocation or gene transfer. They are also stable to processing and resistant to treatment by the gastric environment, making them an ideal inclusion in functional foods and nutraceuticals (Piqué et al. 2019).

### 7.2 Guts–Brain Axis Interaction: Evidence From Postbiotic Studies

In preclinical models, several animal studies have shown the supportive role of heat‐killed microbial strains on neurobehavioral parameters. (Maehata et al. 2019) revealed that the administration of heat‐killed *Lactobacillus helveticus* MCC1848 greatly alleviated the levels of anxiety and depression behaviors in mice. The results obtained with the behavioral tests demonstrated improvement, and molecular analysis revealed a shift in gene expression in one of the critical areas related to emotion regulation, that is, the nucleus accumbens. Similarly, *Enterococcus faecalis* EC‐12, heat‐killed in C57BL/6J mice, found a decrease in anxiety‐related behavior that contributes to alterations in the expression of neurotransmitter receptor genes in the prefrontal cortex and enrichment of the beneficial gut bacteria like *Butyricicoccus* (Kambe et al. 2020).

ADR‐159, a heat‐inactivated combination *of L. fermentum/L. delbrueckii* postbiotic was introduced to the diet of mice and the levels of corticosterone were found to be decreased and the microbiome diversity change, indicating an effect on the HPA axis (Warda et al. 2019). Similarly, another study reported that the same treatment in corticosterone‐treated mice confirmed high brain‐derived neurotrophic factor (BDNF) and serotonin levels, which are accompanied by enhanced mood behaviors (Wei et al. 2019). A study of a stress‐induced depression model showed that *L. plantarum DP189* increased dopamine levels and reduced the pro‐inflammatory cytokines, meaning anti‐inflammatory properties and regulation via monoamine. Individual‐specific strain effects (Zhao et al. 2020). *L. plantarum* strains have had anxiolytic effects in their postbiotic responses, indicating a need to select specific strains as well (Barros‐Santos et al. 2020). *Bifidobacterium breve* CCFM1025 increased short‐chain fatty acids (SCFA) production and ameliorated brain‐derived neurotrophic factor (BDNF) expression in chronically stressed mice, with amelioration of gastrointestinal and nervous systems (Tian et al. 2020).

Human clinical trials also offer substantial evidence supporting postbiotic use for mood regulation. In a randomized controlled trial, it was found that heat‐inactivated *L. gasseri* CP2305 reduced anxiety and improved sleep in 60 chronically stressed students, possibly via adjustment of n‐valeric acid and normalization of the amount of *Bifidobacterium* (Nishida et al. 2019). Research shows that in healthy adults, supplementation with heat‐killed *L. paracasei* MCC1849 changed the mood and enhanced resilience to upper respiratory infection, which are accompanied by higher salivary IgA and beneficial intestinal changes (Murata et al. 2018). Research data shows that individuals suffering from recurrent respiratory infections have benefited from supplementation with polyvalent microbial bacterial lysates. Further, it has been found that heat‐killed *M. manresensis* has been shown to increase T cells in adult patients suffering from tuberculosis (Zhao et al. 2024).

In patients with major depressive disorder (MDD), *L. plantarum* 299v supplementation results in a reduction of kynurenine in serum and an increase in delayed recall, which is an indication of restored tryptophan metabolism (Rudzki et al. 2019). It was found that *Bacillus coagulans* MTCC 5856 enhanced depressive symptoms in persons with IBS and MDD, thus confirming the therapeutic promise of the community‐brain interventions (Majeed et al. 2018). In a pilot study involving MDD patients, researchers observed better mood and sleep after treatment with *L. helveticus* R0052 and *B. longum* R0175 (Wallace and Milev 2021). Similarly, another study revealed that a probiotic‐prebiotic combination lowered cortisol and increased tryptophan ratios, proving that the HPA axis is a target of postbiotic therapies. This evidence is further reinforced by budding research (Kazemi et al. 2019).

Emerging studies continue to strengthen this evidence. The postbiotics obtained by *Lactobacillus plantarum* ameliorated the adverse effect of *Salmonella* on cognition and alleviated neuroinflammation by preserving SCFA levels in a mouse model (Wu et al. 2022). The results demonstrated a marked elevation of 5‐HT, BDNF, GABA, and dopamine, which support the multi‐modal neuroprotective activity of postbiotics. Additionally, a comprehensive review again confirmed the strong relationship between gut dysbiosis and depression, and retained the therapeutic changes that postbiotics may bring to the vagus nerve, cytokine signaling, and neurotransmitter production (Mohan et al. 2023).

### 7.3 Postbiotics and Their Benefits in Metabolic Disorders

Postbiotics do not need to be living and colonizable; unlike probiotics, this more easily makes them safer and more convenient for those with damaged gut integrity or dysbiosis. They consist of short‐chain fatty acids (SCFAs), peptides, exopolysaccharides, teichoic acids, and other bioactive components of microbial metabolism or lysis. These substances affect host health in various ways, such as reinforcing the gut barrier, regulating metabolism, and modulating immunity (Chen et al. 2024). A double‐blind randomized clinical trial shows that supplementation with pasteurized *A. muciniphila* in obese, insulin‐resistant patients improved insulin sensitivity and reduced insulinemia and plasma total cholesterol. This supports the effective use of postbiotics in humans (Zhao et al. 2024).

Obesity is an epidemic problem all over the world, which is caused by the consumption of too much energy in the body as a result of an excessive amount of body fat. However, recent reports have attributed great significance to the pivotal regulation of host metabolism by the gut microbiota and their metabolic wastes (Martorell et al. 2021; Miyamoto et al. 2019; Tseng and Wu 2019; Wang et al. 2018). Among these, SCFAs, in combination, especially acetate, propionate, and butyrate, are successfully used as postbiotics with a substantial anti‐obesity effect. They are synthesized through fermentation of dietary fiber by microbes and have their effects working through G‐protein‐coupled receptors, including GPCR41 and GPCR43, affecting lipid metabolism, insulin signaling, and energy expenditure (Yao et al. 2020; Zhang et al. 2021). It is important to note that the activation of GPCR43 suppresses the process of lipogenesis and stimulates fat catabolism, which facilitates the decrease in the volume of body fat (Zhang et al. 2021).

SCFAs also regulate the release of GI hormones such as peptide YY (PYY) and glucagon‐like peptide‐1 (GLP‐1) that inhibit appetite and increase insulin sensitivity as well. It is believed that acetate infusing the distal colon of obese individuals produces increased fat oxidation by associating with PYY (van der Beek et al. 2016). Similarly, propionate has also shown its capacity to promote PYY and GLP‐1 release in colon cell lines in humans (Pingitore 2017). Propionate and butyrate animal models have been demonstrated to decrease gluconeogenesis in the liver and lipogenesis, as well as subsequently activating gluconeogenesis in the lower GIT, respectively, leading to reduced adiposity. Other postbiotic metabolites of microbial metabolism, vanillic acid and KetoA were also reported to enhance thermogenesis and activate brown adipose tissue (Vallianou et al. 2020). Supplementation with Uro‐A and Uro‐B in rats under a high‐fat diet (HFD) led to a decrease in body weight and cholesterol, as well as the LDL‐C, whereas concentrations of HDL‐C were elevated and helpful microbes such as Bacteroidetes and Firmicutes, which prevented obesity‐induced dysbiosis of the gut (Abdulrahman et al. 2021).

Similarly, another study showed that urolithin A not only reduces the size of adipocytes and body weight in mice with obesity but also enhances energy dissipation and induces browning of the white adipose tissue (WAT) and thermogenesis in the brown adipose tissue (BAT), resulting in better lipid levels and reduced inflammatory cytokines in the liver. Butyrate is one of the short‐chain fatty acids that appear to have anti‐obesity ramifications (Xia et al. 2020).

Butyrate supplementation elevated fatty acid oxidation and activated thermogenic genes like PGC‐1 alpha and UCP‐1 in BAT to finally improve insulin sensitivity and less adiposity (Amiri et al. 2023). Similarly, further research showed that bacteriocin PJ4 from *L. helveticus* significantly reduced adipocyte size, serum lipids, and inflammatory cytokines in obese mice, while modulating gut microbiota composition (Bai et al. 2020). Heat‐inactivated *Ligilactobacillus* salivarius strain 189 ingestion significantly reduced weight gain and improved gut microbiota profiles in a pig model, specifically by improving the population of beneficial *Lentisphaerae* and declining *Prevotella*. These microbial shifts were associated with enhanced lipid metabolism pathways (Kim et al. 2021). K8 cell lysates inhibited lipid buildup in adipocytes by suppressing the JAK/STAT pathway and activating the AMPK signaling pathway. (Lim et al. 2022). These findings were further extended by demonstrating that both live and heat‐treated K8 reduced TG levels and downregulated adipogenic markers such as PPARγ and C/EBPα, confirming the strain's anti‐obesity properties in vitro and in vivo (Jang et al. 2023).

Likewise, butyrate derivative hydroxyl‐methyl butyrate has proven to be promising toward the reduction of sarcopenia, a widespread complication that could be present in elderly diabetic patients. It is a potentially useful adjuvant therapy in the aging population with T2DM (Maykish and Sikalidis 2020). Clinically, the benefits of postbiotics have been substantiated by several randomized trials. Pasteurized *Akkermansia muciniphila* was added to an overweight adult group; it sensitized insulin sensitivity by 28.6% and lowered total cholesterol. These were attributed to an increase in mucin production, regeneration of the gut barrier integrity, and inhibition of inflammation mediated by TLR4 (Depommier et al. 2019). It was further identified that Amuc_1100, a membrane protein from *A. muciniphila* , replicated these metabolic benefits in mice (Plovier et al. 2017). Similarly, it was found that there is an enhanced development of glucose homeostasis in obese patients with diabetes when using ileo‐colonic delivery of conjugated bile acid, as it permits the secretion of GLP‐1 through a TGR5 receptor (Calderon et al. 2020). Postbiotics, which are rich in exopolysaccharides and SCFAs from *B. breve* 091109, caused a marked rise in GLP‐1 and PYY production and, resultantly, an increase in insulin secretion and HbA1c levels (Amobonye et al. 2025).

Heat‐inactivated *Lactobacillus* strains substantially decreased fasting glucose and LDL cholesterol, augmented levels of HDL, and improved insulin sensitivity. All these benefits were mainly due to the activation of AMP‐activated protein kinase (AMPK) through SCFA, inhibition of liver glucose production, and stimulation of anorexigenic intestinal hormones (Zhang et al. 2024). Butyrate especially has the potential to increase peripheral insulin sensitivity in lean individuals, though there is less of a response in individuals with metabolic syndrome (Bouter et al. 2018).

Postbiotics can trigger the release of tight junction proteins such as occludin and claudin, as well as promote goblet cell mucin secretion and anti‐inflammatory responses due to increased regulatory T cells and M2 macrophages. These measures lower intestinal permeability and chronic inflammation that are critical in the onset of insulin resistance and metabolic syndrome (Chen et al. 2024). Formulations such as Para probiotics and fermented infant formula (FIF), which are postbiotics, even push the therapeutic potential further. Inactivated microbial cells known as Para probiotics have immune‐modulating and metabolic value without the risk of infection (Vallianou et al. 2020).

### 7.4 Anti‐Oxidant Activity

Postbiotics are thought to have antioxidant properties because they contain both enzymatic and non‐enzymatic antioxidants. In addition to the non‐enzymatic antioxidants are phenolic substances, glutathione, folic acid, short‐chain fatty acids (SCFAs), peptides, and extracellular polysaccharides (EPS). Additionally, glutathione peroxidase, catalase, and superoxide dismutase are examples of enzymatic antioxidants that have antioxidant properties (Hosseinzadeh et al. 2025). Further postbiotic enzymes glutathione peroxidase (GSH‐px), SOD, nicotinamide adenine dinucleotide (NADH)‐oxidase, and NADH‐peroxidase possess antioxidant activity. Cell‐free extracts (postbiotic) of *Lacticaseibacillus paracasei* show lipolytic activity at 2395 μg/g, and the occurrence of antioxidant enzymes SOD, CAT, and GSH‐px was also seen during biochemical profiling (Amobonye et al. 2025). Studies show that postbiotics have a positive impact on CAT activity, GSH activity, and T‐AOC activity, and there was a significant reduction in MDA content in broilers (Humam et al. 2020). In chronic inflammatory states, antioxidant enzymes such as glutathione peroxidase and superoxide dismutase limit damage to tissues by scavenging free radicals. Furthermore, vitamins function as enzymatic cofactors necessary for cellular maintenance, and organic acids, bacteriocins, and biosurfactants have antimicrobial actions by reducing pH or rupturing pathogen membranes (Bueno et al. 2025).

The protective effects of postbiotics *L. plantarum* RG14, RG11, and TL1 were assessed. Of these, the RG14 strain exhibited the highest antioxidant activity in the ABTS radical scavenging experiment and a higher DPPH value, all of which indicate stronger antioxidant potential (Izuddin et al. 2020).

The study demonstrated that the exopolysaccharide obtained from *Lactococcus lactis* subsp. *Lactis* is a postbiotic with an antioxidant property by raising the levels of antioxidant enzymes (such as glutathione peroxidase, catalase, and superoxide dismutase) and lowering the levels of lipid peroxidation (such as malondialdehyde) in mouse livers and serum (Blazheva et al. 2022). *L. casei* had the strongest antibacterial properties overall. However, in the current investigation, *L. plantarum* had mild antioxidant characteristics but was unable to stop any harmful bacteria (Yaqub et al. 2021). On peripheral blood mononuclear cells, postbiotics produced from *Bifidum* and *Bifidum* exhibit significant anti‐inflammatory properties. Accordingly, the application of postbiotics decreased the production of IL‐8, a cytokine associated with inflammation, in intestinal epithelial cells and increased the output of IL‐10, an anti‐inflammatory cytokine that is essential for controlling inflammatory reactions in peripheral blood mononuclear cells (Abbasi et al. 2022). *L. plantarum‐derived* extracellular vesicles promote human monocytic THP1 differentiation toward an anti‐inflammatory phenotype, which promotes an anti‐inflammatory cytokine profile in human skin tissues, thus signifying a potential role in mitigating hyperinflammatory skin conditions (Kim et al. 2020).

### 7.5 Postbiotics and Their Role in the Prevention and Treatment of Anemia

Among the most well‐documented postbiotic agents are vitamins, short‐chain fatty acids (SCFAs), and amino acids such as tryptophan (Trp). Folic acid (FA) is perhaps the most established postbiotic with direct implications in hematopoiesis. It is crucial for DNA synthesis, amino acid metabolism, and red blood cell formation. FA deficiency often manifests as megaloblastic anemia and is a common nutritional concern in populations with inadequate dietary intake or increased physiological demands. Importantly, FA is not only obtained from diet but can also be synthesized by commensal intestinal bacteria. High folate‐producing strains include *Bifidobacterium bifidum* and *B. longum subsp. infantis*, while moderate‐to‐low‐level production is observed in *B. breve* and *B. adolescentis* (Pompei et al. 2007). Clinical studies demonstrated elevated fecal FA concentrations in patients colonized by these bifidobacteria (Strozzi and Mogna 2008). Moreover, *L. acidophilus* has also shown to be associated with reduced anemia prevalence in human populations (Kaur et al. 2021).

Butyrate and propionate have been shown to increase the expression of hypoxia‐inducible factor 2α (HIF2α), a master regulator of genes involved in iron absorption, such as ferroprotein and divalent metal transporter 1 (Das et al. 2020). However, findings across animal studies are heterogeneous. Study noted increased SCFA levels in mice with anemia (Soriano‐Lerma et al. 2022), while reported reduced SCFA‐producing bacteria in iron‐deficient rats. In human infants with IDA, a reduction in butyrate‐producing genera like *Roseburia* and *Coprococcus* was observed, though fecal butyrate levels remained unchanged, suggesting complex compensatory mechanisms in SCFA metabolism (Dostal et al. 2013). Serum Trp levels positively correlate with hemoglobin and ferritin concentrations. A 10% rise in serum Trp is associated with a 1.59% increase in hemoglobin and a 6.45% increase in ferritin, highlighting its influence on both iron storage and utilization (Wenninger et al. 2019).

### 7.6 Postbiotics and Their Mineral‐Modulating Benefits

The gut microbiota has a central role in modulating mineral absorption in the host, with specific microbial metabolites such as short‐chain fatty acids (SCFAs) improving mineral solubility and uptake. Postbiotics can interact with this microbiota–mineral axis in several beneficial ways. For instance, studies have shown that selenium or selenium/zinc‐enriched postbiotics significantly modulate the intestinal microbiome, leading to enhanced mineral bioavailability compared to their inorganic counterparts (Kang et al. 2020; Yang et al. 2021).

Because minerals incorporated in postbiotic matrices are integrated into bioactive exopolysaccharides and cell wall components, they are more efficiently absorbed. This may protect the minerals from interactions that impede absorption in the gastrointestinal tract. Selenocompounds, for instance, that remain enslaved in the exopolysaccharide matrix of *Lactobacillus* species have demonstrated improved bioavailability and support better antioxidant defense through incorporation into host selenoproteins such as thioredoxin reductases and glutathione peroxidases (Mörschbächer et al. 2018). Furthermore, the immunomodulatory benefits of postbiotics are amplified by the inclusion of trace elements. Zinc, for example, not only enhances the stability of postbiotic preparations but also improves their functional capacity in modulating immune responses and oxidative stress. Zinc‐enriched postbiotic strains like *Lactobacillus plantarum* have demonstrated increased resistance to gastrointestinal stressors and higher antioxidant activity (Meng et al. 2022).

Animal studies offer compelling evidence supporting the superiority of mineral‐enriched postbiotics. In broiler chickens, a diet supplemented with selenium‐enriched *Bacillus subtilis* improved gut microbiota diversity and body weight more significantly than inorganic selenium or probiotic‐only diets (Yang et al. 2021). Similarly, in murine models, selenium/zinc‐enriched *Lactobacillus plantarum* altered gut microbial composition, increased beneficial bacterial abundance, and elevated blood mineral levels, leading to improved antioxidant defenses (Kang et al. 2020). It has been seen that postbiotics enhanced with zinc or selenium increase the expression of host antioxidant enzymes and decrease oxidative stress indicators. This directly affects diseases like metabolic syndrome, cancer, and infectious diseases like SARS‐CoV‐2 that are marked by inflammation or immunological dysregulation (de Brito et al. 2021). Health benefits conferred by postbiotics are further summarized in Figure 3.

**FIGURE 3:**
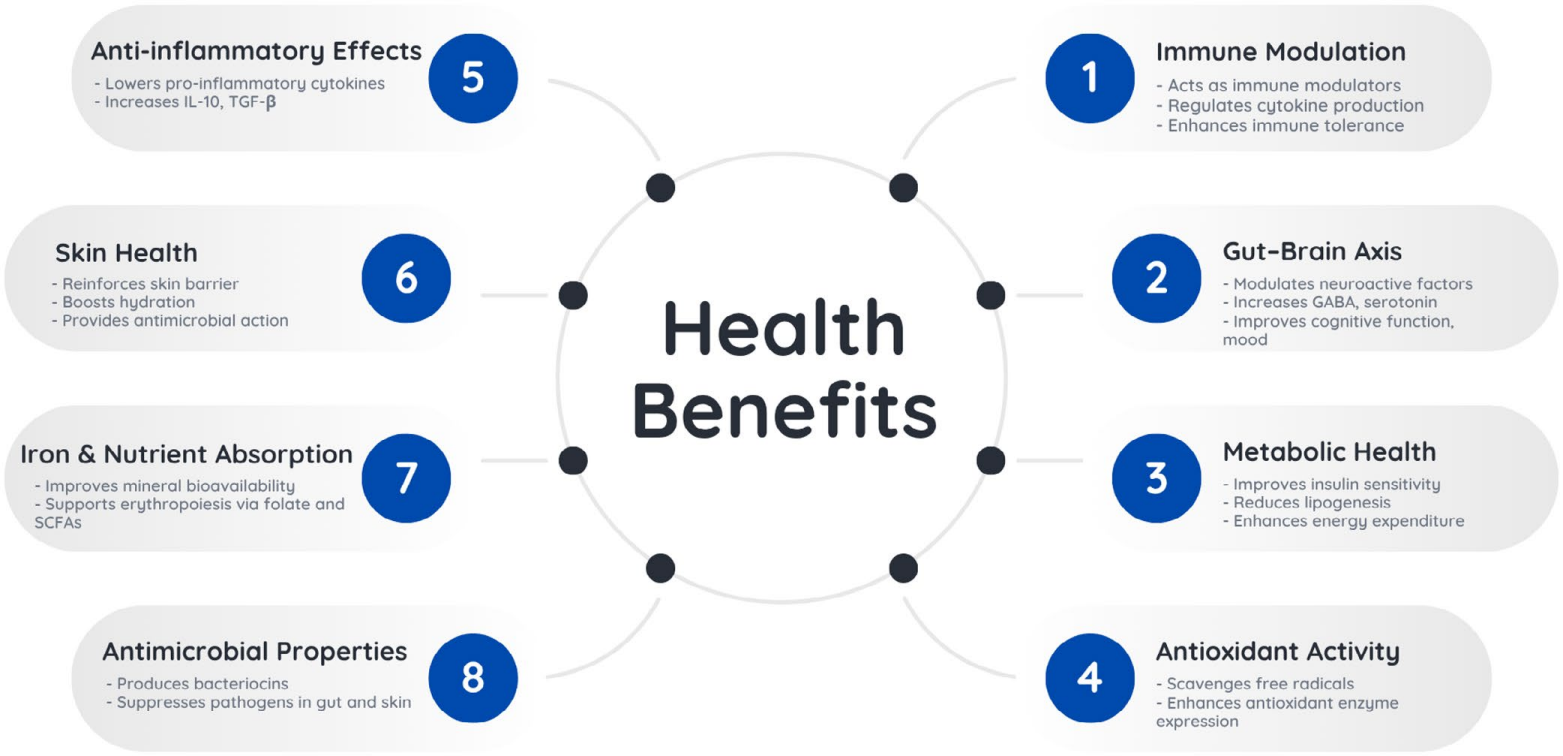
Overview of health benefits, including antimicrobial, anti‐inflammatory, immunomodulatory, and metabolic regulatory effects associated with postbiotics.

## 8 Emerging Applications

### 8.1 Mental Health

Numerious studies have reported improvemnets in cortisol levels, sleep latency, and sleep length following pr, pre, or postbiotic therapy; however, no discernible changes in stress, anxiety or sleep were dtetected (Haarhuis et al. 2022). It is found in research that post‐culture media obtained by lactic acid bacteria, which include *Bifidobacteria and Lactobacilli*, possess the ability to protect neural cells from the cytotoxic effects of in vitro neurodegenerative disease inducers, such as rotenone and MPP*+. Bifidobacterium longum‐*derived postbiotics are also found to increase brain‐derived neurotrophic factor (BDNF) expression in SH‐SY5Y cells stimulated with corticosterone (Bashir et al. 2025).

Literature indicates that postbiotics generated from *Lactobacillus plantarum* (LP) significantly reduce neuroinflammation and brain damage. According to behavior tests, the metabolites socially shielded animals from the memory loss, anxiety, and depressive‐like behaviors brought on by ST. There have also been reports of changes in neuroactive substances such as 5‐hydroxytryptamine, gamma‐aminobutyric acid, dopamine, acetylcholine, neurotrophic factor derived from brain, and neuropeptide Y. (Wu et al. 2022). It is found that taking the postbiotic *L. gasseri* CP2305 (1 × 1010 cfu/day) decreased STAI scores after the intervention when compared to a placebo; it had no impact on salivary cortisol levels, but there was a decline in CgA (Haarhuis et al. 2022).

Changes in the mRNA expression of GABAA and GABAB receptors reduce depressive behavior. GABA, which is released by *Lacticaseibacillus rhamnosus* and *Lacticaseibacillus casei*, has an anti‐depressive impact. It goes into the brain through the neural route, which regulates the GABAergic system and HPA axis. *Levilactobacillus brevis* secretes GABA, which promotes restful sleep and prevents depressive symptoms (Pandey et al. 2022a). Receptors associated with G‐proteins allow short‐chain fatty acids (SCFAs), such as butyrate, propionate, and acetate, to improve mental condition and cognition by influencing the function of the central nervous system's 5‐HT system and BDNF expression (Dziedzic et al. 2024; Pandey et al. 2022a). According to research on *Lactobacillus reuteri*, probiotics may utilize a bacterial‐derived histidine deacetylase (Postbiotic) to make histamine from dietary histidine in the colonic lumen. This histamine then acts on H2 receptors to decrease inflammation in the colon, lessen damage to the colonic epithelium, and stop the decline of BDNF, a measure of mental health (Ma et al. 2024; Pandey et al. 2022b).

Extracellular vehicles (EVs) generated from *Lactobacillus* have been shown to restore the alterations in neurotrophic factor expression brought on by stress. EVs from *L. plantarum* and *L. paracasei* prevent stress‐induced reductions in proBDNF expression while increasing the expression of BDNF and other neurotrophic factors. These alleviated the behavioral outcomes of stressed mice and were achieved by the control of Sirt1 and Mecp2 in mice (Baek et al. 2024). In rodent models, research indicates that *Enterococcus faecalis* and *Lactobacillus helveticus* that have been heat‐killed have anxiolytic and antidepressant‐like properties. Additionally, supplementing with *Lactobacillus paracasei* MCC1849 reduces stress‐induced decline in elevated moods in healthy people, and heat‐inactivated *L. gasseri* CP2305 lessens anxiety and sleep disruption in young adults who encounter stress (Chudzik et al. 2021).

### 8.2 Sports Nutrition and Muscle Health

A study comparing live and heat‐killed *Lacticaseibacillus paracasei* PS23 in healthy adults revealed that while both types of cells enhanced endurance during exercise and facilitated recovery from muscle damage more quickly, heat‐killed cells significantly impacted fatigue index, strength recovery, and vertical jump height more than live cells (Kerksick et al. 2024). Consumption of heat‐killed HKB‐3, or *Bifidobacterium breve* B‐3, following two or four weeks, postbiotics enhance the distribution of oxidative fibers in the gastrocnemius muscle and boost strength in the grip, which eventually encourages muscular hypertrophy and modification (Yang et al. 2024). Protein proportions from *L. delbrueckii* CIDCA 133 postbiotics encourage mucosal healing and strengthen the intestinal epithelial barrier, while heat‐processed *Lactiplantibacillus plantarum* postbiotics demonstrate improvements in muscle mass and work by means of anti‐inflammatory and anti‐catabolic mechanisms (Bueno et al. 2025). In the study, the anti‐sarcopenic effects of postbiotics (WDK) derived from polyphenol‐rich melon peel extract (*Cucumis melo L. var. makuwa*, KEE) and whey with *Lentilactobacillus kefiri* DH5 (DH5) improved the atrophy of C2C12 cells caused by palmitate, re‐established the length and diameter of myotubes, and increased the amount of expression of myogenic genes such as Atrogin‐1, Igf‐1, and MyoD (Han et al. 2023). According to a study, a postbiotic made of heat‐killed *Lactiplantibacillus plantarum* KM‐2, its metabolites, and an excipient (soybean powder) improved muscle mass, performance of muscles, and serum lactate dehydrogenase values in DEX‐treated mice by decreasing the expression of genes linked to skeletal muscle degradation (Atrogin‐1 and MuRF1) while increasing the anabolic phosphorylation of FoxO3a, Akt, and mTOR in C2C12 cells (Jeong et al. 2024). It is seen that the gut microbiota core member *Parabacteroides distasonis* (Pd), which is abundant in centenarians, increases the strength of the skeletal muscles in mice aged 18 and 26 months. According to proteomic studies of the gastrocnemius muscle, Pd raises the expression of proteins linked to mitochondrial preservation and fitness (Sauma et al. 2025).

### 8.3 Commercial Products

Commercially available products like Hylak Forte and Colibiogen oral are designed to enhance gut health. A cell‐free filtrate obtained from *Escherichia coli* cultures (strain Laves 1931) that contains a variety of chemical products of metabolism, including amino acids, peptides, polysaccharides, and fatty acids, is the basis for the commercial product Colibiogen oral (Scott et al. 2022). The primary ingredients of the commercial postbiotic product ColibiogenVR are polysaccharides, fatty acids, peptides, and amino acids. Colitis and overall health were improved in a mouse model when Colibiogen was consumed under control (Abbasi et al. 2022). To lower the pH of the GI tract and prevent harmful bacterial growth, Hylak Forte is a postbiotic that has the natural metabolites of four particular bacterial strains: *Enterococcus faecalis, L. acidophilus, Lactobacillus helveticus*, and *Escherichia coli* . It is also recommended for symptoms like flatulence, diarrhea, and constipation that are linked to bacterial disparities in the GI tract (Patil et al. 2019).

Many commercial pharmaceutical products, including Lacteol (inactivated *Lactobacillus* sp.*), Nyaditum resea* (inactivated Mycobacterium), and Cytoflora (cw containing *Lactobacillus* and *Bifidobacterium* sp.), have received FDA approval for use as immunomodulators, supplements, and infection risk reduction measures (Aggarwal et al. 2022). The microbes in the fermented infant formula, specifically *S. thermophilus* 065 and *Bifidobacterium breve* C50, are rendered inactive by spray drying following fermentation. Fermentation products and inert microorganisms are present in newborn formula. Its safety and postbiotic qualities, such as modification of the gut microbiota to resemble that of breastfed infants, were demonstrated in several clinical trials conducted on children (Vinderola, Sanders, and Salminen 2022). Alcohol‐inactivated *L. fermentum* CNCM MA65/4E‐1b plus is another pharmaceutical product that contains treating children's diarrhea with *L. delbrueckii subsp. delbrueckii* CNCM MA65/4E‐2z and their fermentation metabolites (Vinderola et al. 2023).

Heat‐inactivated *Bifidobacterium bifidum* MIMBb75 is the basis for a CE‐marked medical device used to manage the symptoms of irritable bowel syndrome that is marketed in Europe (Andresen et al. 2020). Figure 4 shows various available commercial products made by using the postbiotics.

**FIGURE 4:**
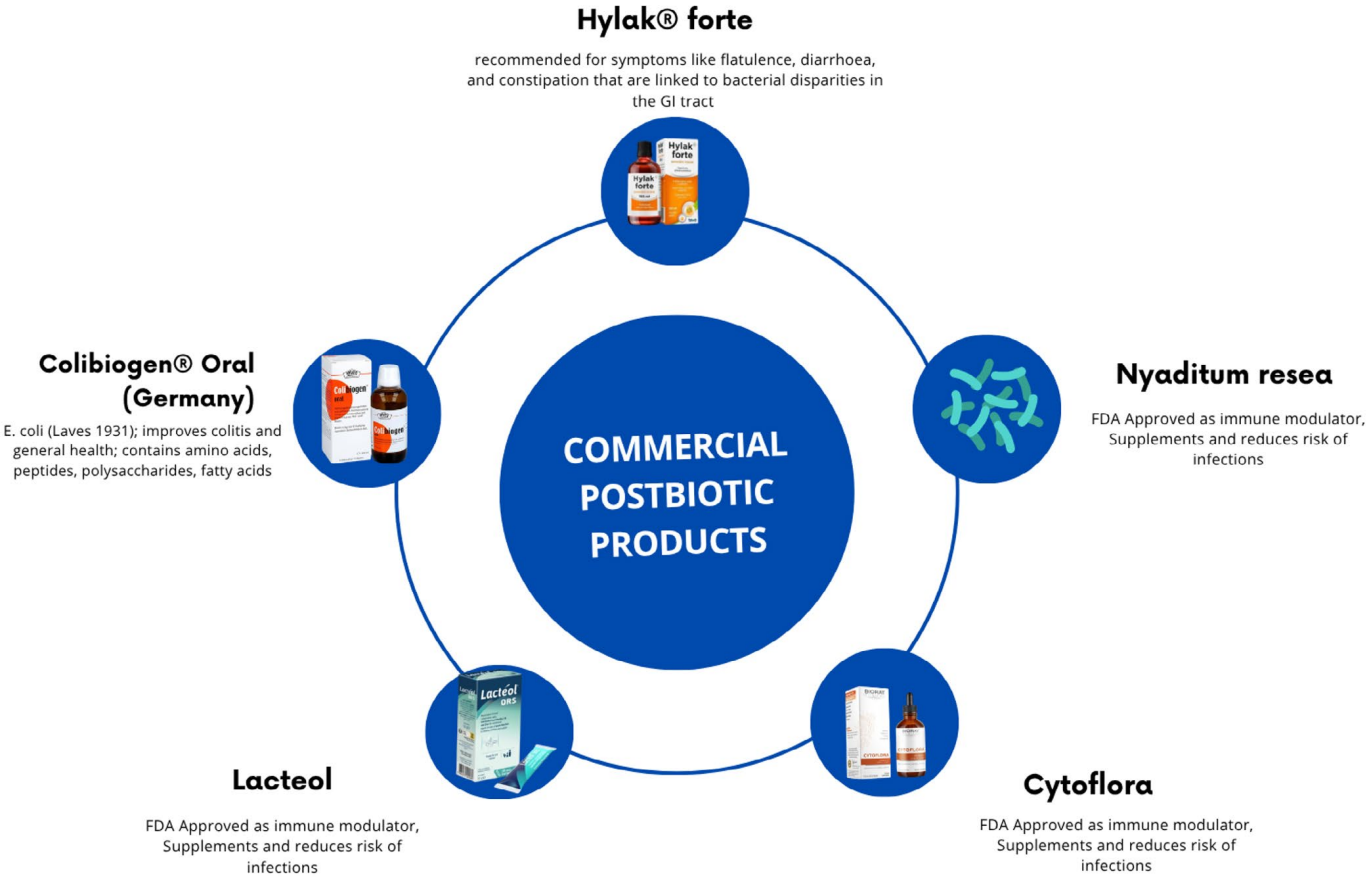
Commercial application in food, nutraceutical, and therapeutic sectors of postbiotic‐based products.

### 8.4 Other Benefits

Postbiotics play a role in eliminating antinutritional factors, such as phytase, an enzyme obtained from *Lactobacillus* spp., which reduces phytic acid content. Tannins and oxalates are degraded by oxalate decarboxylase and tannase enzymes produced by microbes. Twenty‐two strains of *Lactobacillus* show antinutritional metabolism properties (Khani et al. 2025).

In the bread industry, *Thermoascus aurantiacus* CBMAI‐derived xylanase hydrolyzes non‐starch polysaccharides, which is found to enhance dough rheological properties and bread volume (Asqardokht‐Aliabadi et al. 2025). Extracts containing postbiotic (PBEs), including cell‐free supernatant (CFS) and exopolysaccharide (EPS) fractions, which are obtained from three probiotic strains such as *Lactiplantibacillus plantarum* UTNGt2, *Lactococcus lactis* UTNGt28, and *Weissella cibaria* UTNGt21O, proved to be promising multifunctional antimicrobial agents for food preservation and have been validated for their safety in food applications (Molina et al. 2025).

Use of postbiotics in animal production has become a major research focus. Addition of high‐temperature dried *Lactobacillus plantarum* (strains 22F, 25F, etc.) and *Pediococcus acidilactici* 72 N in piglet diets improved feed conversion, increased fecal *Lactobacillus* counts, and reduced the quantity of harmful bacteria such as *Enterobacter*. Supplementation of heat‐killed *Enterococcus faecalis strain EC‐*12 causes a rise in total IgA in cecal digestive fluid, and total IgG signifies its immune‐boosting application in poultry (Yue et al. 2025).

## 9 Limitation

Despite a lot of literature stating benefits associated with the postbiotics production and usage, several limitations exist. The most obvious is the imbalance in the evidence itself. A large amount of data has been gathered from in vitro systems and rodent models. Data from the human trials that do exist are typically small, short, single‐centre, and heterogeneous in the preparations. The metabolic findings illustrate this well: the pasteurized *Akkermansia muciniphila* study that reported improved insulin sensitivity was an exploratory proof‐of‐concept trial in a modest number of participants (Depommier et al. 2019). Animal‐to‐human extrapolation is a recurring weakness across the gut–brain and anti‐obesity literature.

A second limitation is the variability between preparations. “Postbiotic” covers everything from a single purified metabolite to a crude heat‐killed whole‐cell lysate, and the inactivation method heat, sonication, high pressure, UV, solvent, or chemical treatment materially changes what ends up in the final product and how bioactive it is (Aguilar‐Toalá et al. 2020). Two products described by the same strain name can therefore differ substantially in composition and effect. This heterogeneity makes findings hard to compare across studies, frustrates dose–response analysis, and is one of the main obstacles to the standardization that the field openly acknowledges it lacks (Vinderola et al. 2025).

## 10 Future Perspectives and Knowledge Gaps

The most pressing need is standardization. There is a need to define postbiotics based on the level of composition of the relevant bioactive fractions and not just by strain name. More work is needed on quantification methods, such as flow cytometry for intact inactivated cells alongside chromatographic and spectrometric profiling of metabolites (Vinderola et al. 2025), which points the way, but consensus reference methods and reporting standards are still missing. A related gap is the near‐total absence of dose–response and long‐term safety data in humans; most trials report a single dose over a few weeks.

Technologically, the interesting frontier is moving from harvesting by‐products to designing them. Precision‐fermentation and integrated engineering approaches that tune the strain, medium, and processing to enrich a target compound are beginning to appear, and could turn postbiotic manufacture into a controllable, reproducible process rather than an incidental one. Computational and AI‐assisted screening predicting which strains and conditions yield which bioactives, and matching those to a desired physiological effect, is a natural complement and would help close the gap between the production side and the clinical side that this review has repeatedly noted.

There is also a strong sustainability and circular‐economy argument that remains under‐exploited. Several of the most promising preparations are produced from food‐industry side‐streams: cheese whey. Systematic evaluation of agri‐food by‐products as fermentation substrates could improve both the economics and the environmental footprint of production and deserves more attention than it currently receives.

Furthermore, postbiotics fit naturally into precision and personalized nutrition. Because their effects clearly depend on host status, for example the lean‐versus‐metabolic‐syndrome difference in butyrate response. Which requires the human trials, biomarkers, and stratification tools. The unresolved questions can be summarized simply: which specific molecules drive which effects, at what dose, in whom, and produced how; and until those are answered strain by strain, much of the field's promise remains to be confirmed.

## 11 Conclusion

During the last decade, postbiotics have moved from being leftovers of fermentation to being treated as a defined class of bioactive preparation. This review clearly shows that postbiotics are more stable, easier to store and transport, and free of the translocation and gene‐transfer risks that occasionally complicate probiotic use. They possess metabolites and cell structures that perform biological activities. Data were gathered on use of postbiotics for immune health, gut health promotion, metabolic health, antioxidant defense, anemia prevention, and mineral status improvement. Postbiotics constitute short‐chain fatty acids, cell‐wall components, secreted peptides, exopolysaccharides, and vitamins and act on receptor signaling, epithelial integrity, and immune tone and produce their subsequent response.

This review highlights the role of different postbiotic components and their respective health benefits. The data on production and characterization show various inactivation methods, media, and processing techniques in postbiotic production and their detrimental effect on the final bioactive profile. Moreover, health evidence in human trials suggests the strongest role in allergy, mood, and early metabolic work, and the weakest claims rest on single strains tested only in cells or rodents.

Postbiotics are a real advance in microbiome‐based nutrition and therapeutics, not a repackaging of probiotics, and several are already in commercial and clinical use. But the gap between their apparent potential and what has actually been demonstrated in well‐designed human studies is still wide. Closing it will depend less on discovering new effects than on discipline: standardized, well‐characterized preparations; transparent reporting of production and dose; preparation‐specific safety and allergenicity data; and trials large and long enough to support the claims being made. If the field meets those requirements, postbiotics are well placed to become a durable component of functional foods and precision nutrition rather than a passing trend.

## Author Contributions

**Zoha Wahid:** conceptualization, methodology, writing – original draft. **Abdul Momin Rizwan Ahmad:** conceptualization, methodology, supervision, writing – review and editing. **Farheen Zulfiqar:** methodology, writing – original draft. **Syed Hassan Bin Usman Shah:** methodology, writing – review and editing. **Nilab Mohammadi:** conceptualization, writing – review and editing.

## Funding

The authors have nothing to report.

## Disclosure

Generative AI Statement: No generative artificial intelligence (AI) tools were used in the preparation of this manuscript.

## Ethics Statement

The authors have nothing to report.

## Consent

The authors have nothing to report.

## Conflicts of Interest

The authors declare no conflicts of interest.

## Supporting information


**Table S1:** Literature search strategy, including databases, search string, Boolean logic, filters, and eligibility criteria.
**Table S2:** Major postbiotic types, representative microbial sources, and principal reported functions.
**Table S3:** Health effects of postbiotics by system: proposed mechanisms, level of evidence, and representative studies. Level of evidence is indicated as in vitro, animal, or human to make the current maturity of each area explicit.

## Data Availability

The authors have nothing to report.
