# Supplementary material for: Postbiotics Unveiled: The Next Emerging Trend in Functional Foods
Source: Food Sci Nutr. 2026 Oct 14;14(10):e72485. doi: 10.1002/fsn3.72485 (PMC13668483; doi:10.1002/fsn3.72485)
Supplement: Supplementary file 1 [file FSN3-14-e72485-s001.docx]

**Supplementary Tables for Review and Publication**

**Supplementary Table 1.** Literature search strategy, including databases, search string, Boolean logic, filters, and eligibility criteria.

| **Parameter** | **Details** |
| --- | --- |
| Databases searched | PubMed; Google Scholar (supplemented by backward reference-list searching of key reviews and consensus statements) |
| Timeframe | Inception to [month/year]; search last updated [month/year], emphasis on 2015–present, with older foundational papers retained where relevant. |
| Intervention keywords | postbiotic*; paraprobiotic; parabiotic*; biogenics; metabiotic; microbiota metabolites; microbial metabolites; cell-free supernatant*; heat-killed bacteria; inactivated probiotic; tyndallized bacteria; ghost probiotic |
| Context / outcome keywords | functional food*; health benefit*; gut microbiota; immunomodulation; short-chain fatty acid*; SCFA; gut–brain axis; metabolic disorder*; obesity; type 2 diabetes; antioxidant; production; standardization; regulation |
| Boolean structure | (intervention terms joined by OR) AND (context/outcome terms joined by OR). Truncation (*) used for word-ending variants; quotation marks for exact phrases. |
| Representative full string | (“postbiotic*” OR “paraprobiotic*” OR “biogenics” OR “metabiotic*” OR “microbiota metabolites” OR “cell-free supernatant*” OR “heat-killed bacteria” OR “inactivated probiotic*”) AND (“functional food*” OR “health benefit*” OR “gut microbiota” OR “immunomodulation” OR “short-chain fatty acid*” OR “gut–brain axis” OR “metabolic disorder*” OR “production” OR “standardization”) |
| Filters applied | Language: English. Publication type: peer-reviewed journal articles. For Table 1 (production data) only: restricted to original research / experimental articles (reviews excluded). |
| Inclusion criteria | English-language, peer-reviewed; addressing definition, production, characterisation, mechanism, or health effects of postbiotics/paraprobiotics/inactivated cells/cell-free fractions; in vitro, animal, or human studies; human and animal nutrition contexts. |
| Exclusion criteria | Non-English; abstracts, preprints, theses, editorials or opinion pieces without primary data; studies of live probiotics only (no postbiotic component); duplicates; for Table 1, review articles as a source of production data. |
| Synthesis method | Narrative, thematic synthesis by application area. No meta-analysis. Qualitative weighting of evidence (human trials and reproducible animal studies weighted above isolated in vitro findings); no formal risk-of-bias tool applied. |

**Supplementary Table 2.** Major postbiotic types, representative microbial sources, and principal reported functions.

| **Postbiotic type** | **Representative source(s)** | **Principal reported function(s)** | **Selected evidence** |
| --- | --- | --- | --- |
| Short-chain fatty acids (acetate, propionate, butyrate) | Fibre fermentation by Lactobacillus, Bifidobacterium, Faecalibacterium spp. | Epithelial barrier support; Treg induction and HDAC inhibition; appetite and glucose regulation via GLP-1/PYY | Parada Venegas et al., 2019; Zheng et al., 2017; van der Beek et al., 2016 |
| Cell-wall components (teichoic acids, peptidoglycan, SLPs) | L. rhamnosus GG; L. acidophilus; L. plantarum | TLR/NOD-mediated immunomodulation; dendritic-cell and Treg signalling | Konstantinov et al., 2008; Behzadi et al., 2021; Amobonye et al., 2025 |
| Secreted proteins / peptides (p40, p75, HM0539, bacteriocins) | L. rhamnosus GG; various LAB | EGFR-mediated tight-junction protection; antimicrobial activity; SIgA induction | Bingöl et al., 2024; Sarkar & Mandal, 2016 |
| Exopolysaccharides (EPS) | Lactococcus lactis; L. plantarum; B. animalis subsp. lactis HN019 | Antioxidant enzyme induction; anti-inflammatory and gut-barrier effects | Blazheva et al., 2022; D’ambrosio et al., 2024 |
| Polyphenol-derived metabolites (urolithins, equol, enterolignans) | Gut microbial transformation of dietary polyphenols | Adipose thermogenesis / browning; lipid and cholesterol modulation | Xia et al., 2020; Abdulrahman et al., 2021; Cortés-Martín et al., 2020 |
| Vitamins and cofactors (folate, B12, vitamin K) | B. bifidum; B. longum subsp. infantis; L. acidophilus | Haematopoiesis; DNA synthesis; enzymatic cofactor supply | Pompei et al., 2007; Strozzi & Mogna, 2008 |
| Enzymatic antioxidants (SOD, catalase, GSH-px) | Lacticaseibacillus paracasei; L. plantarum | Free-radical scavenging; reduction of lipid peroxidation (MDA) | Hosseinzadeh et al., 2024; Izuddin et al., 2020 |
| Whole inactivated cells / lysates (paraprobiotics) | Heat-killed L. paracasei, A. muciniphila; bacterial lysates (PMBL) | Immune priming; metabolic and mucosal effects without viability risk | Depommier et al., 2019; Suárez et al., 2020 |
| Extracellular vesicles (EVs) | L. plantarum; L. paracasei | Neurotrophic (BDNF) signalling; anti-inflammatory skin effects | Baek et al., 2024; Chikindas et al., 2026 |

**Supplementary Table 3.** Health effects of postbiotics by system: proposed mechanisms, level of evidence, and representative studies. Level of evidence is indicated as in vitro, animal, or human to make the current maturity of each area explicit.

| **Health domain** | **Key mediators** | **Proposed mechanism** | **Highest level of evidence** | **Representative studies** |
| --- | --- | --- | --- | --- |
| Immune modulation | Teichoic acids, SLPs, SCFAs, IL-10/TGF-β | PRR (TLR/NOD) engagement; Treg induction; barrier reinforcement | Human (allergy, elderly) | Lee et al., 2016; Akatsu, 2021; Roberts et al., 2024 |
| Gut–brain axis | SCFAs, BDNF, GABA, serotonin, HPA-axis modulation | Vagal and neuroendocrine signalling; neurotransmitter and BDNF regulation | Human (small RCTs) | Nishida et al., 2019; Rudzki et al., 2019; Majeed et al., 2018 |
| Metabolic disorders | SCFAs, urolithins, Amuc_1100, GLP-1/PYY | GPR41/43 signalling; improved insulin sensitivity; adipose browning | Human (proof-of-concept) | Depommier et al., 2019; Plovier et al., 2017 |
| Antioxidant activity | SOD, catalase, GSH-px, EPS, phenolics | Enzymatic and non-enzymatic radical scavenging; lower MDA | Animal / in vitro | Blazheva et al., 2022; Izuddin et al., 2020; Humam et al., 2020 |
| Anaemia / haematopoiesis | Folate, SCFAs, tryptophan | Microbial folate synthesis; HIF-2α-linked iron-transporter regulation | Mixed / limited human | Pompei et al., 2007; Wenninger et al., 2019 |
| Mineral modulation | Se/Zn-enriched EPS and cell walls, SCFAs | Enhanced mineral solubility and bioavailability; selenoprotein support | Animal | Kang et al., 2020; J. Yang et al., 2021 |
| Muscle / sports nutrition | Heat-killed cells, peptides, HMB | Anti-catabolic signalling (FoxO3a/Akt/mTOR); oxidative-fibre support | Human (early) / animal | Kerksick et al., 2024; Jeong et al., 2024 |
